# The Psychological, Social and Behavioral Impact of Intravitreal Anti-VEGF Therapy: An Analysis from the ALBATROS Data

**DOI:** 10.3390/jcm12237435

**Published:** 2023-11-30

**Authors:** Christian Wolfram, Norbert Pfeiffer, Tobias Hudde, Alexander Klatt, Birthe Schnegelsberg, Mike Ross, Focke Ziemssen, Alexander K. Schuster

**Affiliations:** 1 Department of Ophthalmology, University Medical Center Hamburg-Eppendorf, Martinistr. 52, 20246 Hamburg, Germany; 2 Department of Ophthalmology, University Medical Center of the Johannes Gutenberg-University Mainz, Langenbeckstr. 1, 55131 Mainz, Germany; norbert.pfeiffer@unimedizin-mainz.de (N.P.); alexander.schuster@unimedizin-mainz.de (A.K.S.); 3 Eye Hospital Wolfsburg-Fallersleben, Am Spieker 10, 38440 Wolfsburg, Germany; tobiashudde@icloud.com; 4 Eye Center Klatt, Henry-Wetjen-Platz 3, 28844 Weyhe, Germany; a.klatt@augenzentrum-klatt.de; 5 Novartis Pharma GmbH, Roonstr. 25, 90429 Nuremberg, Germany; birthe.schnegelsberg@novartis.com (B.S.); mike.ross@novartis.com (M.R.); 6 Department of Ophthalmology, Leipzig University Hospital, University of Leipzig, Liebigstraße 12, Haus 1, 04109 Leipzig, Germany; focke.ziemssen@medizin.uni-leipzig.de; 7 Center for Ophthalmology, Eberhard-Karls-Universität Tübingen, Elfriede-Aulhorn-Str. 7, 72076 Tübingen, Germany

**Keywords:** anti-VEGF, patient perspective, attitude, psychology, health care, fear, vision loss, blindness

## Abstract

Background: Retinal diseases such as neovascular age-related macular degeneration (nAMD), diabetic macular edema (DME), or branch/central retinal vein occlusion (B/CRVO) have significant implications for patients’ social and psychological well-being. The ALBATROS study aimed to assess the care situation of patients who received anti-VEGF (vascular endothelial growth factor) treatment. To gain a comprehensive understanding of patients’ backgrounds and attitudes, we developed an exploratory, structured questionnaire, the Basic Care and Patient Satisfaction Questionnaire (BPZ-9). Methods: The data collection took place at the beginning and after twelve months of anti-VEGF therapy. The BPZ-9 questionnaire comprises nine questions to evaluate patients’ psychological and social situation and satisfaction with treatment. Results: Data were collected from 1478 nAMD (mean 78 years), 445 DME (67 years), 233 BRVO (70 years), and 144 CRVO (71 years) patients at 102 study centers throughout Germany. One in four patients had difficulties walking, and one in five needed an accompanying person for treatment. Anxiety about losing vision was present in three out of four patients at the beginning, and it slightly decreased to two out of three patients over the 12-month treatment period. The distress of having a retinal disease was generally higher than the distress related to the treatment. Most patients reported high treatment satisfaction (73%) and felt well-informed (81%). Conclusions: There is a relevant social and psychological impact related to anti-VEGF treatment. The patients’ perception, attitudes, and commitment need further investigation.

## 1. Introduction

Intravitreal injections are among the most commonly performed procedures in medicine. Their positive impact on preserving vision in retinal diseases such as neovascular age-related macular degeneration (nAMD), diabetic macular edema (DME), and retinal vein occlusions (RVO) has been extensively demonstrated [1,2,3,4]. The widespread use of these injections has contributed to a reduction in blindness and low vision [5,6]. However, individual perspectives on treatment may differ from a population health standpoint, as they require continuous motivation over many years [7,8]. Consequently, non-persistence and non-adherence remain significant challenges that threaten the long-term maintenance of visual function [9]. It is essential to understand patients’ attitudes and commitment to the treatment in order to explain and enhance treatment adherence [10]. Previous publications have revealed substantial disparities in treatment practices between clinical studies and real-life scenarios. Notably, the injection frequency and treatment adherence are often much lower in real-world settings compared to clinical trials [11,12]. As a result, patients’ perspective has garnered increased attention in recent years to gain a deeper understanding of their concerns, relevant barriers, and behavioral aspects.

Retinal diseases and comorbidities have a profound impact on the quality of life (QoL) [13]. Since a significant portion of treated patients are elderly, this target group often suffers from other health problems and polypharmacy and experiences daily limitations even before regular treatment visits, and ocular discomfort introduces additional burdens for both patients and potential caregivers [14]. Questionnaires that focus solely on QoL dimensions do not adequately capture the personal situations and treatment commitments of patients. Therefore, we have developed an exploratory tool aimed at comprehensively elucidating patients’ social, physical, and psychological conditions. This tool should aid in understanding the utilization of care by patients and in potentially identifying internal or external barriers and limitations to therapy.

## 2. Materials and Methods

The exploratory questionnaire was implemented within the multi-centered ALBATROS data collection to monitor the situation of anti-VEGF patients when receiving treatment for neovascular age-related macular degeneration (nAMD), diabetic macula edema (DME), or retinal vein occlusion (RVO) in Germany. The study design and clinical outcomes have been recently reported [15]. One hundred and two study centers participated in the project throughout Germany. Patients were invited to join the data collection before commencing their therapy and were followed-up over the first twelve months of treatment. No specifications regarding anti-VEGF treatment or treatment frequency were given. Only the patients without prior anti-VEGF treatment, aged ≥18 years, and with written informed consent for participation were included in the study.

**Figure 1:**
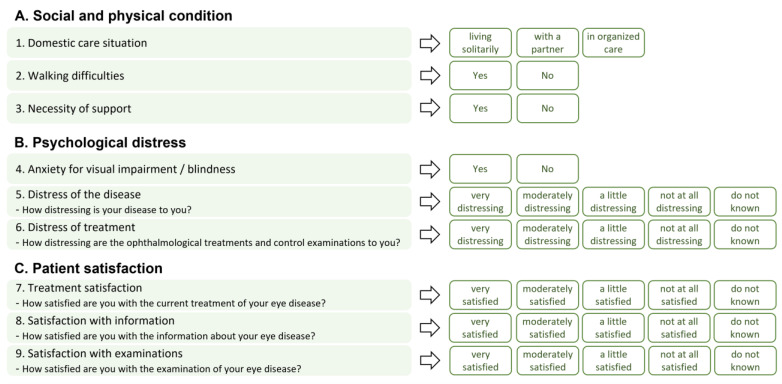
Questions and answer options of the exploratory BPZ-9 questionnaire.

The structured questionnaire about the patients’ situation of basic care and patient satisfaction (in German: “Fragebogen zur Basisversorgung und Patientenzufriedenheit”, BPZ-9) comprised nine questions in three different domains about their social, physical, and psychological conditions. The patients’ distress and satisfaction with the treatment were assessed using a five-point Likert scale. The answers were collected at the baseline and the final study visit (after twelve months). An overview of the questions and answer options is provided in Figure 1.

For this analysis, data from patients who completed the full 12-month observation period were included. Statistical analyses were descriptive, with absolute and relative frequencies, tabulated using SAS Version 9.4 (SAS Institute Inc., Cary, NC, USA).

## 3. Results

In total, 1478 patients with a baseline and a 12-month visit were included in the completed analysis set (CAS) of the ALBATROS data collection. During the 12-month observation period, the patients received on average (mean ± SD) 6.1 ± 3.2 anti-VEGF injections.

The mean (±SD) age of the study population was 74.5 ± 10.9 years, with a 54.9% of female patients. Irrespective of sex, older patients experienced worse visual acuity (Figure S1). Nearly two-thirds of the study’s population (65.2%) were treated for nAMD, with 32.8% receiving bilateral treatment (DME: 9.9%). The demographic parameters varied across different treatment indications, e.g., patients diagnosed with nAMD were generally older and more likely to be female, while patients diagnosed with DME were younger and more likely to be male (Table 1).

**Figure 2:**
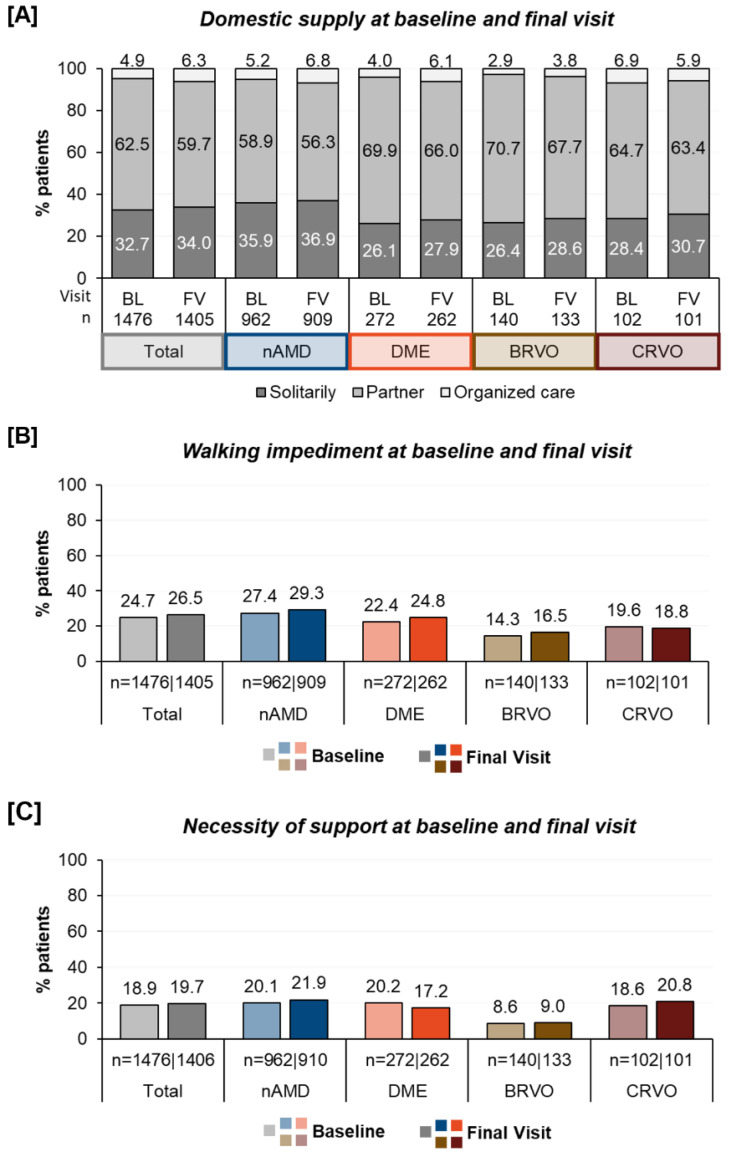
Domestic care situation (**A**), prevalence of walking difficulties (**B**), and need for an accompanying person (**C**) at the baseline and after twelve months of treatment. BL, baseline; and FV, final visit.

Social and physical condition: The living situation of the patients varied significantly. At the baseline, 4.9% of the patients lived in organized care, 62.5% with a partner, and approximately one third (32.7%) lived alone. At the baseline, 24.7% of the patients reported walking impairments and 18.9% reported dependency on an accompanying person. The percentages varied by treatment indication, as demonstrated in Figure 2, and showed that the nAMD patients more often suffered from walking difficulties and required more assistance. In contrast, the BRVO-patients were less often living in organized care and reported a better walking situation and a lesser need for an accompanying person.

After twelve months of treatment, the proportion of individuals in a single household increased from 32.7% to 34.0%, and the percentage of those in organized care rose from 4.9% to 6.3%. Walking difficulties slightly increased from 24.7% to 26.5%, and the need for assistance during anti-VEGF treatment rose from 18.9% to 19.7%. Fewer patients with DME were dependent on support than at the beginning of treatment (20.2% vs. 17.2%).

**Figure 3:**
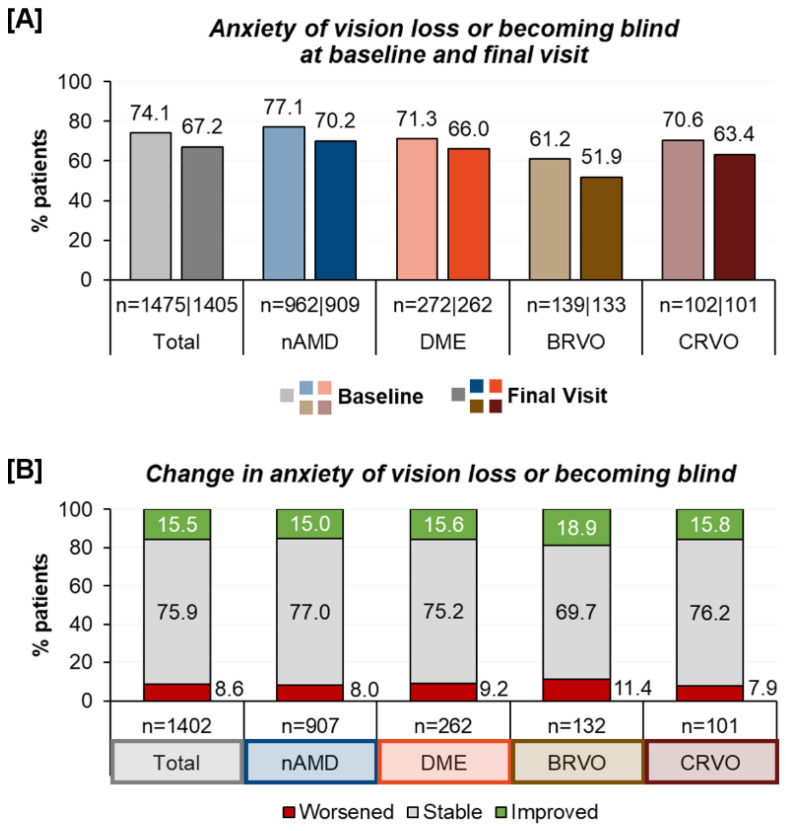
Anxiety of vision loss and blindness (**A**) and change in anxiety from the baseline to the final visit for all treatment indications (**B**).

Psychological distress/Anxiety of vision loss: Prior to the first anti-VEGF therapy, most patients (74.1%) expressed anxiety about developing visual impairment and blindness. This concern remained present in 67.2% of the patients after twelve months. When examining different underlying treatment indications the fear of vision loss was less prominent in the branch retinal vein occlusions (BRVO) patients and more prominent in the nAMD patients. Over the course of one year of treatment, a reduced prevalence of anxiety was observed across all treatment indications (Figure 3). However, while 15.5% had less anxiety about vision loss, a small group (8.6% of patients) reported being more anxious than prior to the anti-VEGF therapy.

Psychological distress/Disease- and treatment-related distress: At the baseline, 71.9% of the patients perceived their disease as moderately to very distressing, while 25.8% perceived it as little or not at all distressing. At the final visit, fewer patients reported disease-related distress, but it was still prevalent in nearly two-thirds of the patients (65.6%). Regarding treatment distress, we found that a majority (52.5%) experienced it as little or not at all distressing, compared to 44.5% who valued treatment as moderately to very distressing. After twelve months, the ratio had not changed substantially (55.1%—“little/not at all distressing” vs. 43.9%—“moderately/very distressing”).

**Figure 4:**
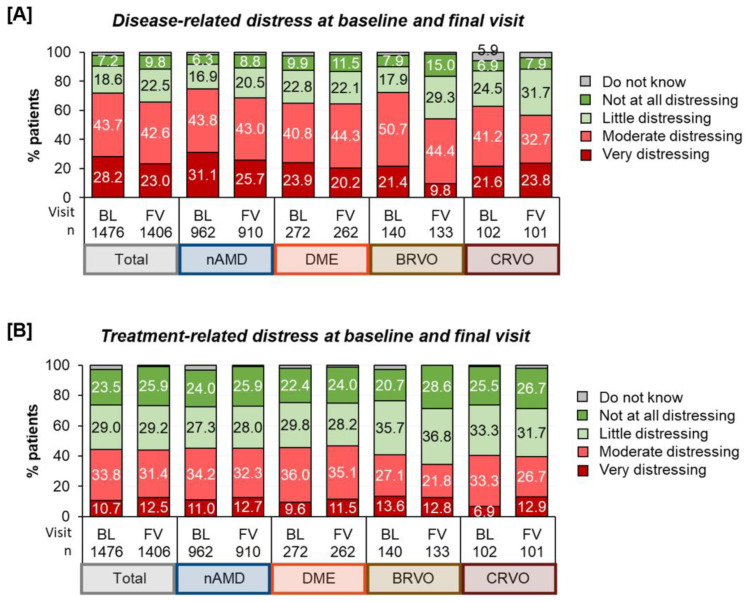
Disease-related distress (**A**) and treatment-related distress (**B**) for all treatment indications. Note: For clarity reasons, labels for values <5% were omitted.

Comparing disease- and treatment-related distress, the patients more frequently struggled with the disease itself than with the treatment (Figure 4).

Regarding differences in disease- and treatment-related distress at the beginning and after twelve months of treatment, we observed that about half of the patients reported similar emotional views. At least, more patients experienced a reduction in disease-related distress than an increase in distress. The results differed by treatment indication, showing that more BRVO patients experienced an improvement than patients with other indications. However, 18.2% of all the patients reported more distress in dealing with the retinal disease, which was also less pronounced among those with BRVO.

**Figure 5:**
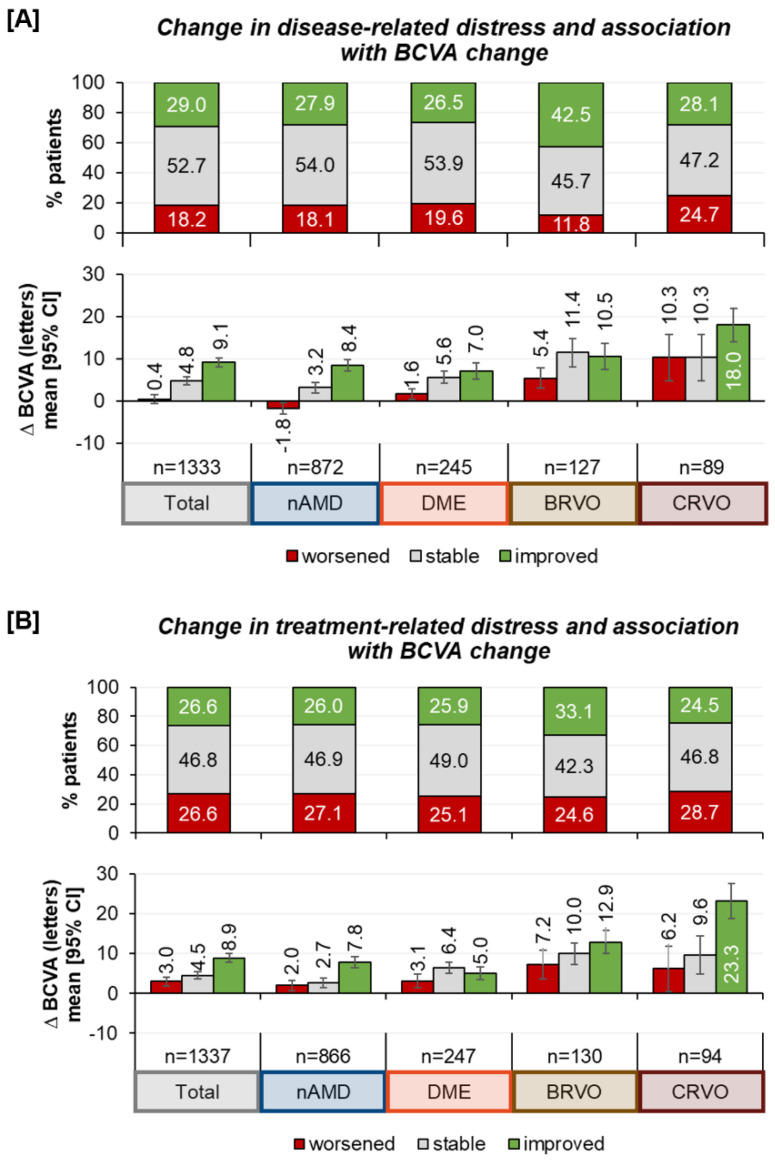
Change from the baseline to the final visit for disease-related distress (**A**) and for treatment-related distress (**B**) in association with the best corrected visual acuity (BCVA, improved: ≥+5 EDTRS letters; stable: >−5 letters and <+5letters; and worsened: ≤−5 letters).

Concerning treatment-related distress, about three out of four patients (73.4%) did not change their perception of treatment distress or even found it improved, with little variation among the different treatment indications (Figure 5B).

Improvements in distress estimation were correlated with changes in the best corrected visual acuity (BCVA), as shown in lower part of the Figure 5A,B. A heat map analyzing the correlation of patients’ disease and treatment distress perception showed no clear correlation of disease and treatment distress, highlighting the value of separately assessing both disease and treatment distress (Figure S2).

**Figure 6:**
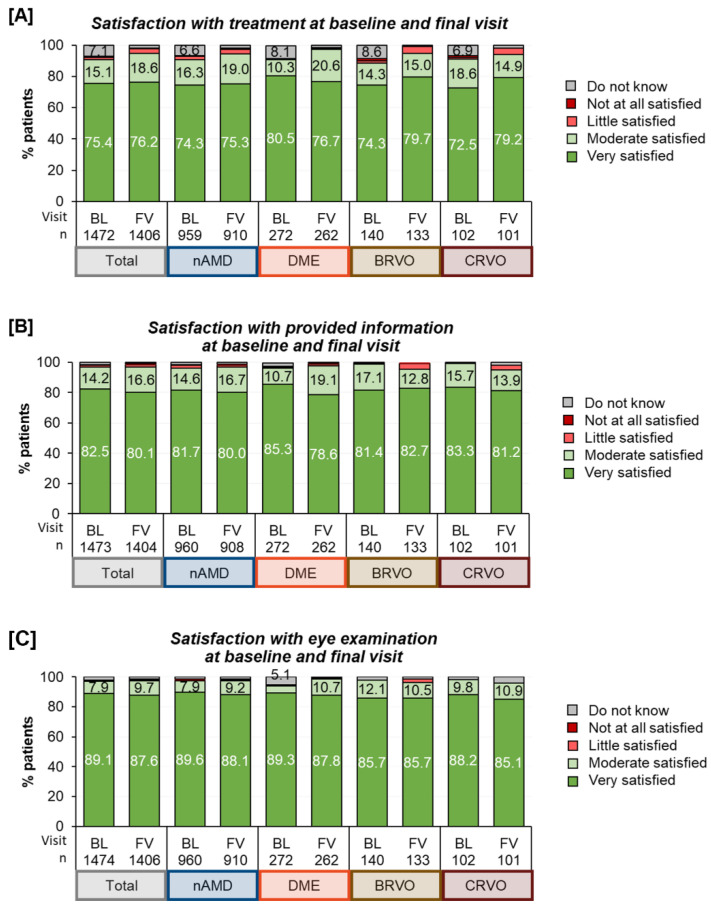
Treatment satisfaction (**A**), satisfaction with eye and control examinations (**B**), and satisfaction with information about disease (**C**) at baseline. Note: For clarity reasons, labels for values <5% were omitted.

Patient Satisfaction: Most patients were generally highly satisfied, regardless of the underlying treatment indication. Their satisfaction varied across different areas, with the highest satisfaction seen for ‘control examinations’ (seven out of eight patients being “very satisfied”), followed by ‘information about the disease’ (four out of five being “very satisfied”) and ‘satisfaction with treatment’ (three of four being “very satisfied”), see Figure 6. Over the course of the twelve months, satisfaction remained generally stable for the patients under study observation. A subgroup analysis revealed that the patients with nine or more injections per year were even more satisfied than those with fewer injections (Figure S3).

## 4. Discussion

The findings from the ALBATROS study provide intriguing insights into the patients’ social, physical and psychological conditions when confronted with a retinal disease and intravitreal anti-VEGF treatment. The extensive size of this study cohort, with data collected from more than one hundred study centers throughout the country, enabled us to capture a representative picture of real-life retinal care in Germany. The broad categories hinted at the variety of practical issues and concerns patients face when receiving injection therapy.

The social backgrounds of anti-VEGF patients are naturally diverse, as reflected in the ALBATROS data, which represented the affected population [16]. Although the vast majority were found to live in their own homes, attention should be given to difficulties or mobility restrictions. Roughly six percent of the patients in our cohort resided in a domestic care situation. More than one-fifth of the patients needed to be accompanied, and even every fourth patient experienced walking difficulties. These facts reveal that logistical issues and limitations may frequently hinder the daily practice of anti-VEGF treatment.

Previous studies have already demonstrated a high need for support. For instance, a survey among nAMD patients reported that 82.1% of patients received assistance from a caregiver [17]. Another study described that support for daily activities was required by 57.8%, even though a high rate of patients (77%) managed to attend medical appointments without assistance [18]. This corroborates our findings that 18.9% of anti-VEGF patients required an accompanying person. The accessibility of care should be closely monitored. Previous surveys suggest that means of transportation do not influence the dependency of affected individuals [14]. Even in cities with good and efficient public transport, visually impaired patients might not manage the injection visit without caregivers.

However, even more important than the issues of transport and mobility, the doctor–patient relationship proved to have a significant impact on the patients [19]. Accompanying persons were able to enhance adherence by overcoming barriers and increasing self-efficacy [20].

The fact that the patients experienced more distress due to their disease than the actual treatment underscores the high psychological burden of having a retinal disease. A review of the psychological impacts of neovascular macular degeneration described that up to 42% of patients showed signs of depression [21]. Other studies reported a prevalence of depression among anti-VEGF patients ranging between 20% and 26% [22] and also among caregivers to AMD-patients, with 24.4% affected [17]. Our findings emphasize the specific impact of anxiety for vision loss as a key psychological element, which was present in as many as 74.1% of the patients at the beginning of their treatment. This high prevalence of anxiety for vision loss confirmed anxiety to be a psychological dimension with a lower threshold than depression, as described in the literature. Assessing anxiety can identify that there is also a fear of the injection itself during treatment and that the efficacy of the treatment is the most important outcome for patients, as previous studies pointed out [23,24]. Patients, therefore, declared their willingness to take certain risks or accept discomfort and inconveniences [25]. Although the possibility of aversions and unconscious preferences cannot be ruled out, the acceptance of intravitreal therapy is generally very high [26], as evidenced by the strongly positive treatment satisfaction outcomes observed in our study.

Our analysis has highlighted the psychological and logistical dimensions of treatment. The literature has described treatment barriers for patients that can be categorized into four groups [19]—tolerability, clinical factors, logistical parameters and human factors—which can all contribute to a low confidence in their therapy and its effects, resulting in the decision to discontinue treatment. The experience of side effects was identified as the most important factor for non-adherence [19]. Previous investigations demonstrated that 28.8% of anti-VEGF patients were lost to follow-up during treatment, defined as a discontinuation of therapy for at least six months [27]. Other studies described that 32% of patients considered interrupting their anti-VEGF therapy [28] and that up to 50% of patients stopped treatment within 24 months [29]. Possible reasons may be a disbelief in the benefit of treatment or a fear of the injection [30]. The follow-up of this study might have been too short to access the full impact of longer treatment durations [31].

Non-adherence is, therefore, a major issue in retinal care, found to be higher in DME than in nAMD patients and other treatment indications [30]. A key role in maintaining adherence was seen in the quality of the relationship between physician and patient [30]. Practical recommendations include better monitoring of the patients’ treatment course to identify interruptions or non-adherence [30] and actively assessing the patients’ expectations regularly along the course of treatment [30]. Even though our analysis focused primarily on those patients who adhered to their therapy, we do observe that psychological and logistical problems are very common among these patients. We can assume that such issues are even more important for those who have difficulties committing to continuous therapy [32]. Future “real-world” research should, therefore, focus even more on those patient groups who do not continue treatment and further investigate behavioral factors.

Our findings were limited because the patients may have responded in an overly positive way and avoided being critical (social desirability bias). In real-world care practice, satisfaction values as demonstrated here may be too high and practical barriers, comorbidities, or polypharmacy may be more prevalent among those who interrupted or even stopped treatment within the first year. Furthermore, our research questions were exploratory, and we cannot provide final information about the validity and reliability of our questionnaire at this point. Further methodological validation is needed to measure the accuracy and consistency of our research questions. The introduction of validated questionnaires can complement established instruments of life-quality and patient-related outcome measures. Future research may also introduce possible means to measure commitment and adherence to ease the delivery of retinal care. As anti-VEGF treatment remains one of the most performed medical procedures, even minor improvements can have a greater impact on the needs of the population.

## 5. Conclusions

In summary, having a retinal disease and undergoing continuous anti-VEGF treatment were shown to be challenging for patients both logistically and psychologically. At the same time, the patients demonstrated high levels of satisfaction with their treatment. Understanding patients’ needs and fears are prerequisites to explaining motivation and commitment to treatment. The high prevalence of fear for vision loss may be a driving factor behind treatment adherence. On the other hand, logistical and psychological barriers might enhance non-adherence, which should be studied and explained further. This link of motivation and behavior, therefore, deserves more investigation and may lead to new insights for encouraging patients and achieving treatment satisfaction. We hope that our exploratory study questions can help widen the knowledge about patients’ social and psychological situations and may serve as a useful complement to other instruments of patient-related outcome measures.

## Figures and Tables

**Table 1:** Demographics and baseline characteristics.

	Total	nAMD	DME	BRVO	CRVO
Demographics and Baseline Characteristics					
Patients, n	1.478	964	272	140	102
Age, years	74.5 ± 10.9	78.3 ± 8.0	65.9 ± 12.3	68.7 ± 11.4	70.4 ± 11.8
Sex, female (%)	54.9	61.1	38.6	52.1	43.1
BCVA study [fellow] eye, letters	56.1 ± 19.6 [65.8 ± 25.0]	54.4 ± 19.6 [62.2 ± 27.2]	65 ± 14.2 [69.2 ± 20.4]	59.1 ± 19.1 [77.2 ± 13.9]	44.9 ± 23.1 [75.3 ± 16.4]
Better eye at baseline is fellow eye, n (%)	1028 (69.6)	643 (66.7)	172 (63.2)	123 (87.9)	90 (88.2)
Neovascular/exudative disease of the partner eye (%)	23.9	32.8	9.9	4.3	3.9

If not otherwise indicated, the data are presented as mean ± SD.

## Data Availability

The data presented in this study are available on request from the corresponding author. The data are not publicly available due to intellectual property reasons.

## Supplementary Materials

The following supporting information can be downloaded at: https://www.mdpi.com/article/10.3390/jcm12237435/s1: Figure S1: Box-Whisker-Plot of worst eye visual acuity vs. age group and sex at baseline. Note: Distribution of worst eye visual acuity (VA): study eye = 69.6%, partner eye = 30.4%. The horizontal lines show the median; the boxes indicate the interquartile range (IQR), with whiskers providing 1.5× IQR and black dots showing outlier values. Figure S2: Heat maps showing the correlation of disease and treatment distress at baseline [A], final visit [B], and the change from baseline to final visit [C] for the overall population. The numbers indicate patients with a distinct combination of distress perception. The shadings show frequencies. The darker the color, the higher the frequency. Figure S3: Satisfaction with treatment, provided information and eye examination stratified by number of received anti-VEGF injections during the observation period for the overall CAS population (total). For clarity reasons labels for values <5% were omitted from the graphs.
