# Supplementary material for: The Psychological, Social and Behavioral Impact of Intravitreal Anti-VEGF Therapy: An Analysis from the ALBATROS Data
Source: J Clin Med. 2023 Nov 30;12(23):7435. doi: 10.3390/jcm12237435 (PMC10707522; doi:10.3390/jcm12237435)

Supplementary Materials:

Figure S1: Box-Whisker-Plot of worst eye visual acuity vs age group and sex at baseline. Note: Distribution of worst eye visual acuity (VA): Study eye=69.6%, Partner eye=30.4%. Horizontal lines show median, boxes indicate the interquartile range (IQR) with whiskers providing 1.5xIQR and black dots showing outlier values.

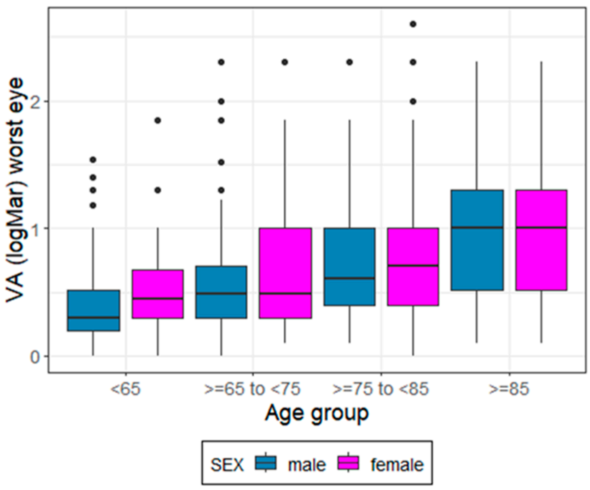

Figure S2: Heat maps showing correlation of disease- and treatment distress at baseline [A], final visit [B] and change from baseline to final visit [C] for the overall population. Numbers indicate patients with a distinct combination of distress perception. Shadings show frequencies. The darker the color, the higher the frequency.

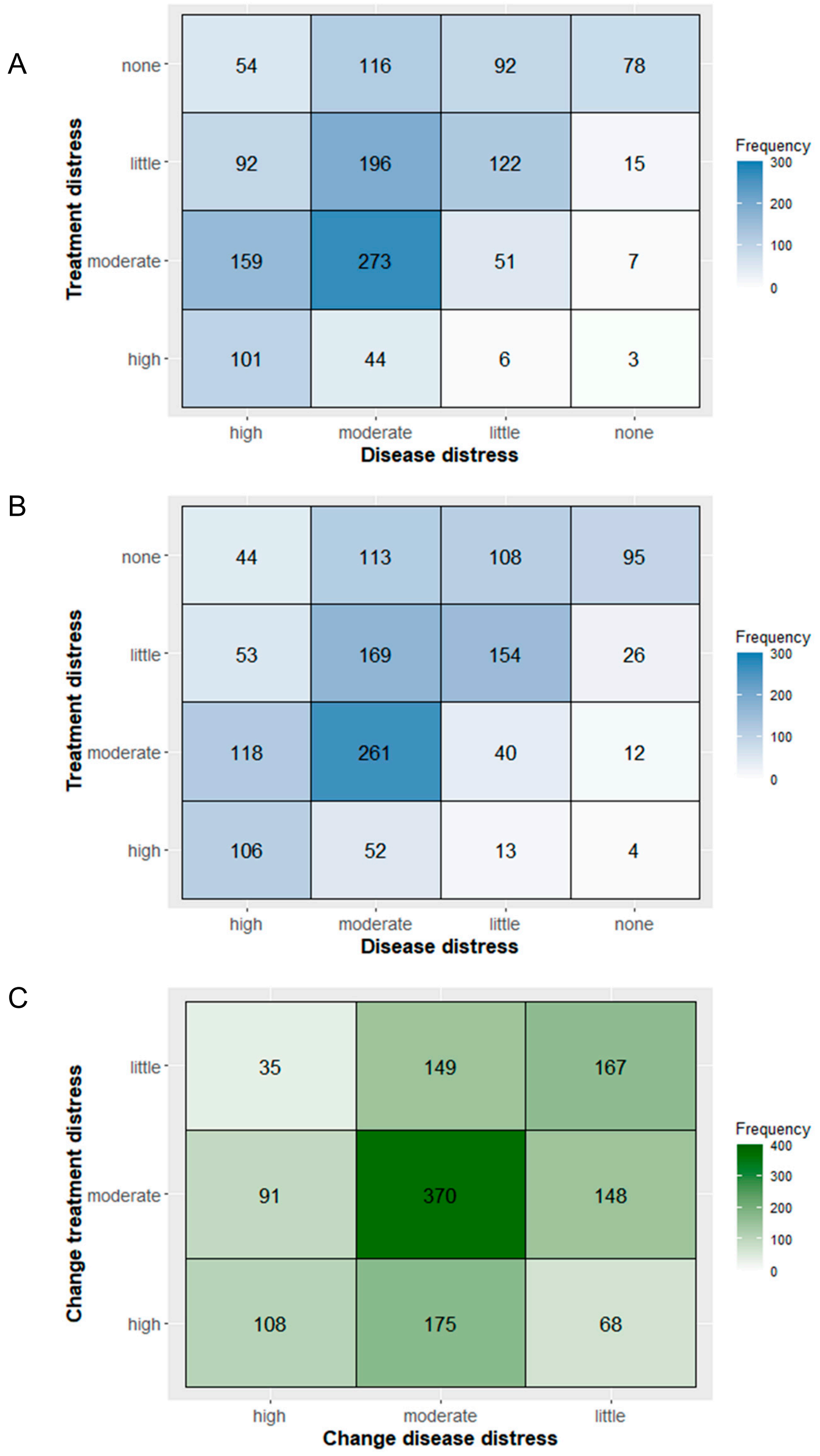

Figure S3: Satisfaction with treatment, provided information and eye examination stratified by number of received anti-VEGF injections during the observation period for the overall CAS population (total). For clarity reasons labels for values <5% were omitted from the graphs.

[A] Satisfaction with treatment

Baseline

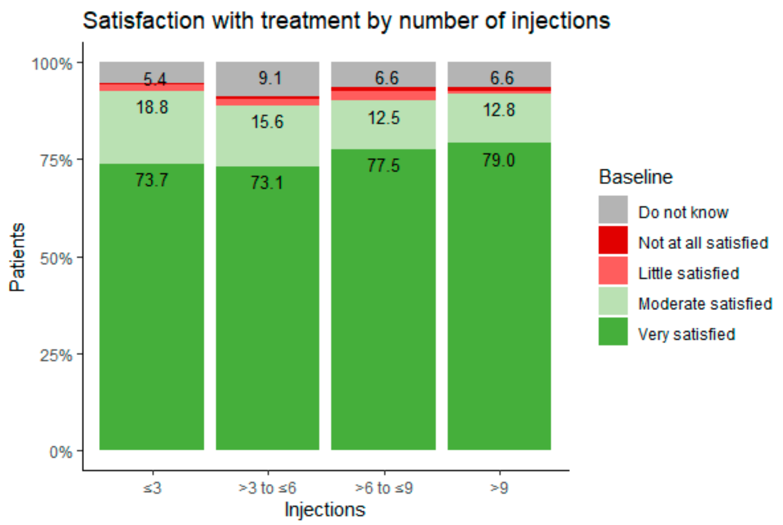

Final Visit

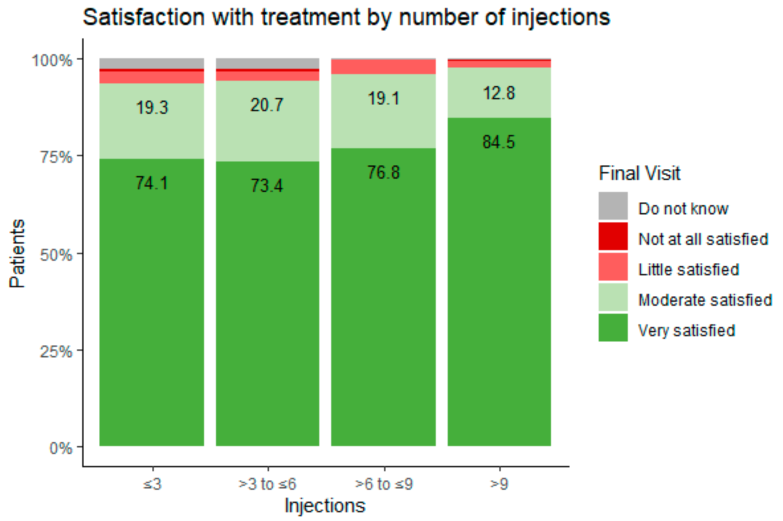

Change from baseline

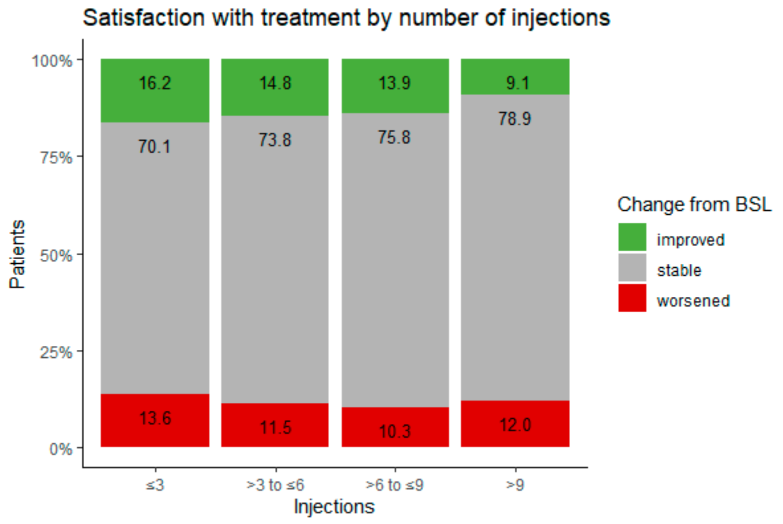

[B] Satisfaction with information

Baseline

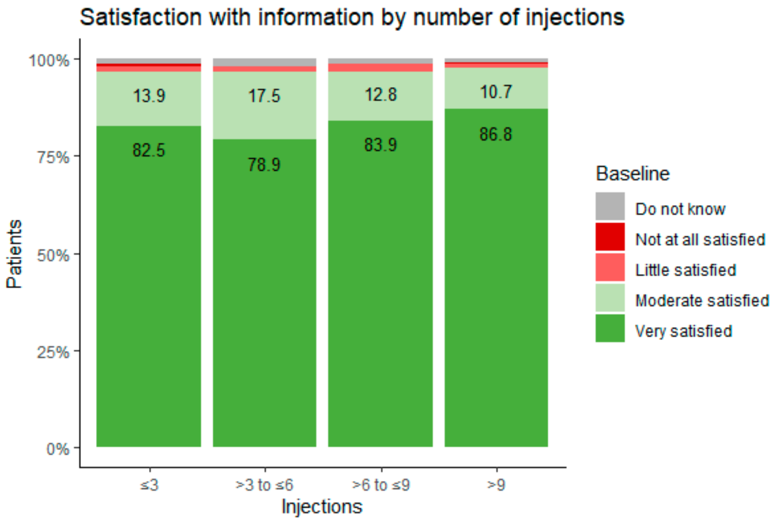

Final Visit

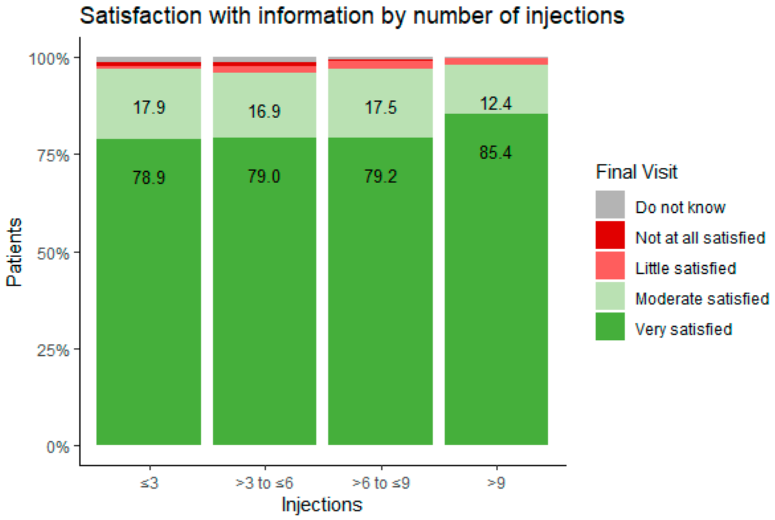

Change from baseline

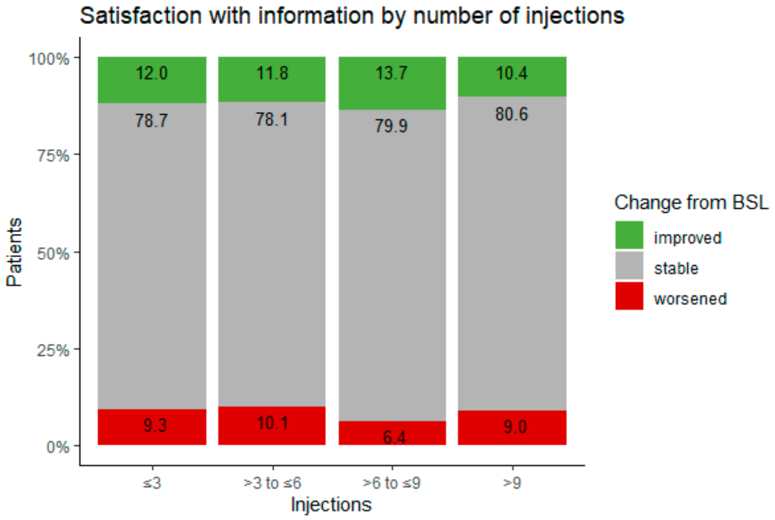

[C] Satisfaction with eye examination

Baseline

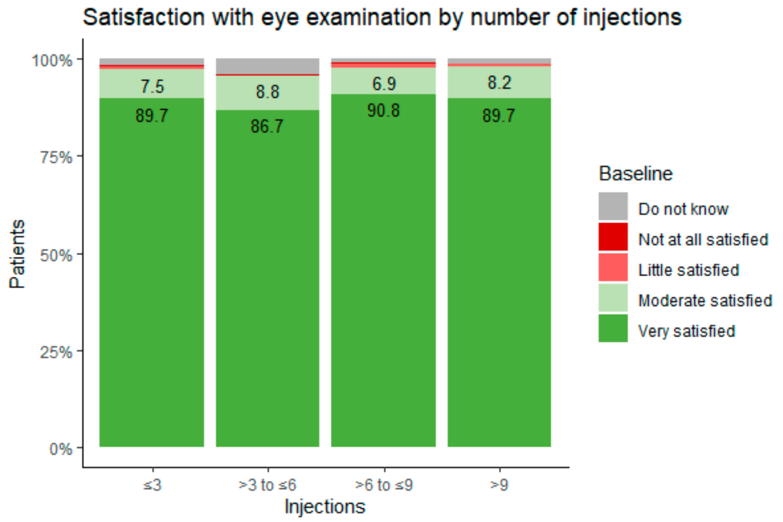

Final Visit

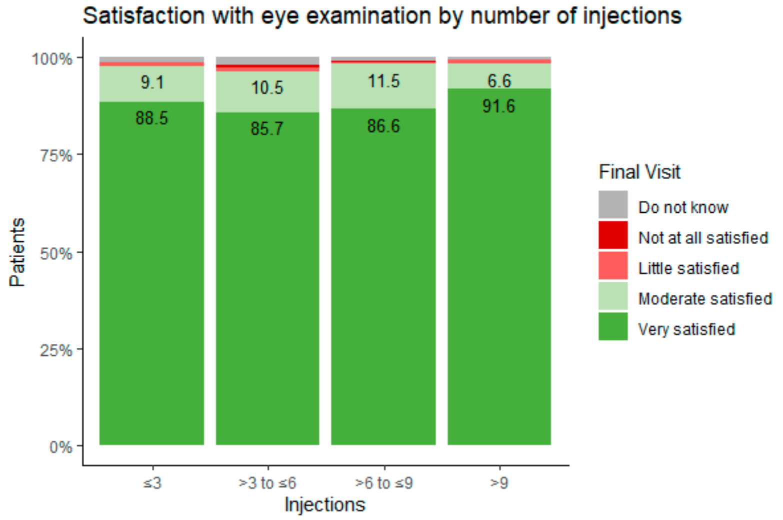

Change from baseline

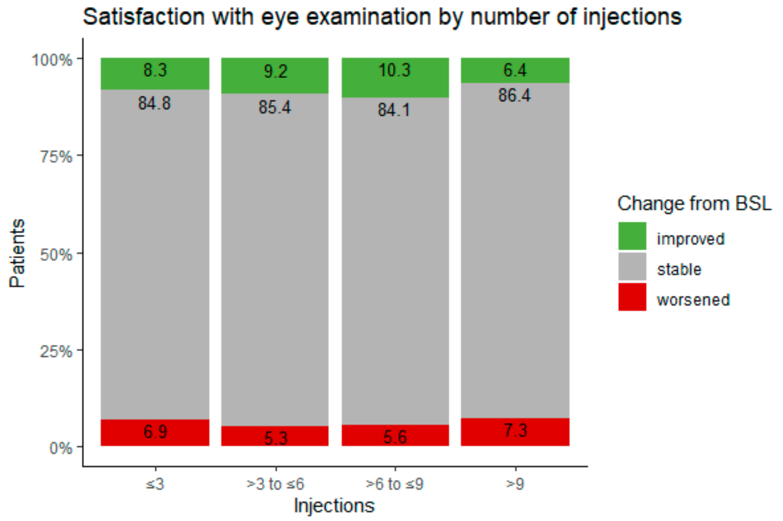

Supplement: Supplementary file 1 [file jcm-12-07435-s001.zip › jcm-2738434-supplementary.pdf]
